# Contrast-enhanced ultrasound of pancreatic melanoma: A case report and literature review

**DOI:** 10.3389/fonc.2022.989638

**Published:** 2022-09-06

**Authors:** Zhiqiang Yuan, Hualin Yan, Wenwu Ling, Yan Luo

**Affiliations:** Department of Ultrasound, West China Hospital, Sichuan University, Chengdu, China

**Keywords:** pancreatic melanoma, pancreatic ductal adenocarcinoma, pancreatic carcinoma, contrast-enhanced ultrasound, enhancement, case report

## Abstract

Melanoma is a malignant tumor that originates from melanocytes, most of which are of cutaneous origin. Most melanomas identified in the pancreas are metastatic, and primary pancreatic melanoma is extremely rare and has rarely been discussed. The correct preoperative diagnosis of pancreatic metastatic melanoma, especially primary melanoma, is challenging. Herein, we report a 43-year-old man who presented to our hospital due to unexplained left abdominal distension and pain. Abdominal ultrasound examination demonstrated multiple space-occupying lesions of the pancreas, and hypoechoic masses partially filled the splenic vein behind the pancreatic body. In the contrast-enhanced ultrasound (CEUS), all of these lesions showed iso-enhancement to slight hypo-enhancement in the arterial phase and hypo-enhancement in the venous phase. Masses in the splenic vein also showed hypo-enhancement. Imaging features suggested that the pancreatic lesions were malignant tumors. The tumor markers carcinoembryonic antigen, carbohydrate antigen 125 and carbohydrate antigen 19-9 were within normal limits. Based on clinical symptoms, imaging findings and incidence of pancreatic tumors, the patient’s clinical diagnosis was pancreatic carcinoma. Surgery was performed for the patient, while postoperative pathology confirmed malignant melanoma of the pancreas. Therefore, it is significant to identify the clinical and imaging manifestations of pancreatic melanoma in order to better manage the disease. Herein, we reported this case and reviewed the literature from 2000 to 2021 on the clinical and imaging features of 26 patients with pancreatic melanoma. It may improve clinicians’ awareness of the clinical and imaging performance of pancreatic melanoma, resulting in improved diagnosis, differential diagnosis, treatment, and outcomes.

## Introduction

Malignant melanoma is a malignant tumor that originates from melanocytes and has a high mortality rate (1). Malignant melanoma of skin origin accounted for 91.2%, and other origin accounted for a small part (2). Most melanomas identified in the pancreas are metastatic, and primary pancreatic melanoma is extremely rare and has rarely been discussed. The clinical symptoms and imaging findings of pancreatic melanoma are not typical. Therefore, the correct preoperative diagnosis of pancreatic metastatic melanoma, especially primary melanoma, is challenging. Preoperative recognition of pancreatic melanoma is significant because of its different treatment and prognosis from other pancreatic malignant tumors. Although imaging features of pancreatic melanoma have been reported in a few cases, no features of pancreatic melanoma have been described through CEUS imaging before. To improve the diagnosis and differential diagnosis of pancreatic melanoma and related imaging findings, we report this unusual case and reviewed the related literature.

## Case presentation

**Figure 1:**
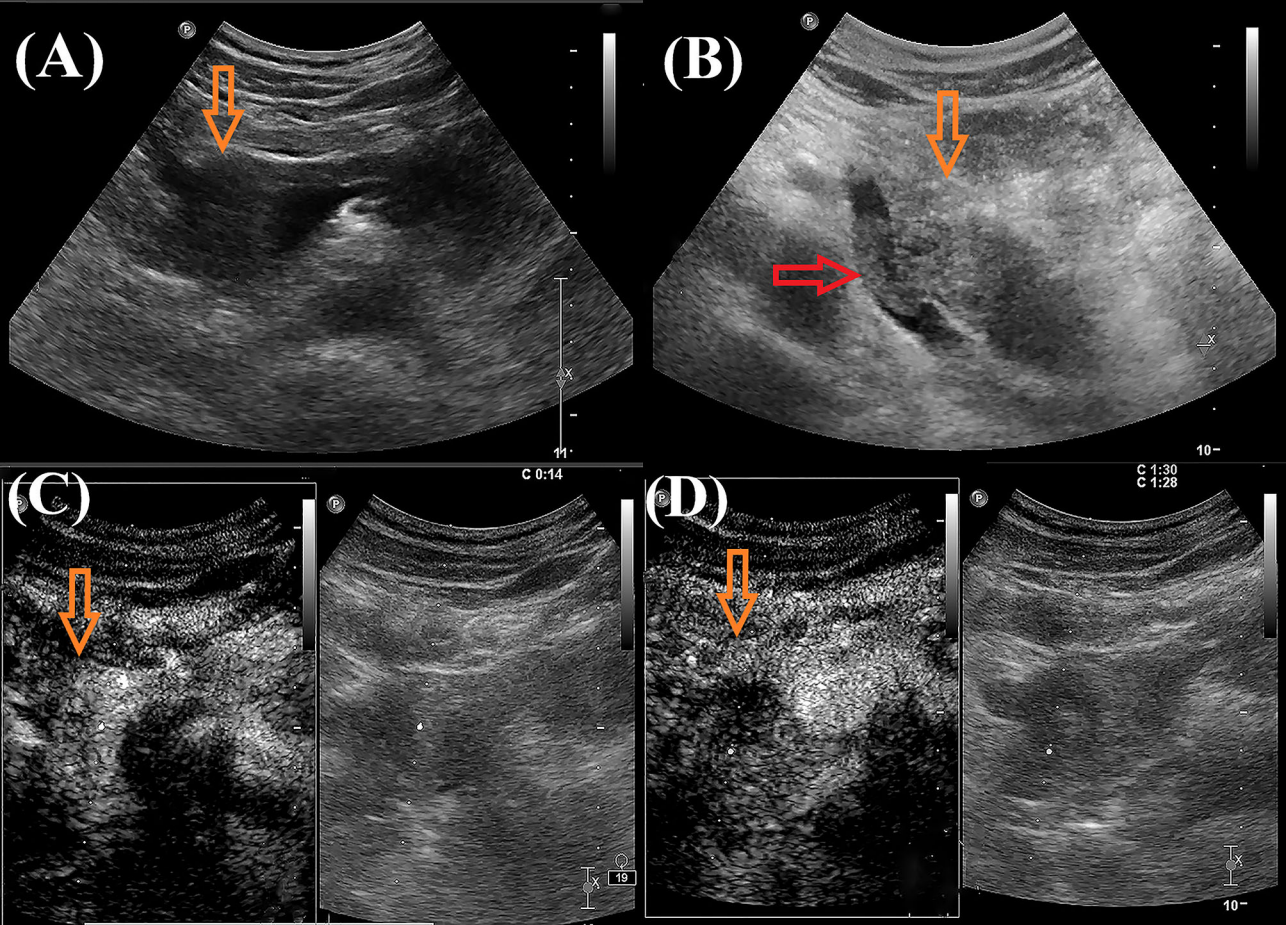
Ultrasound images of the patient. **(A, B)**: Grayscale ultrasound showed hypoechoic mass in the pancreatic parenchyma (orange arrow) and hypoechoic masses partially filled in the splenic vein (red arrow).In the arterial phase, contrast-enhanced US (CEUS) showed iso-enhancement to slight hypo-enhancement [**(C)**, orange arrow)]; In the venous phase, CEUS showed hypo-enhancement [**(D)**, orange arrow].

**Figure 2:**
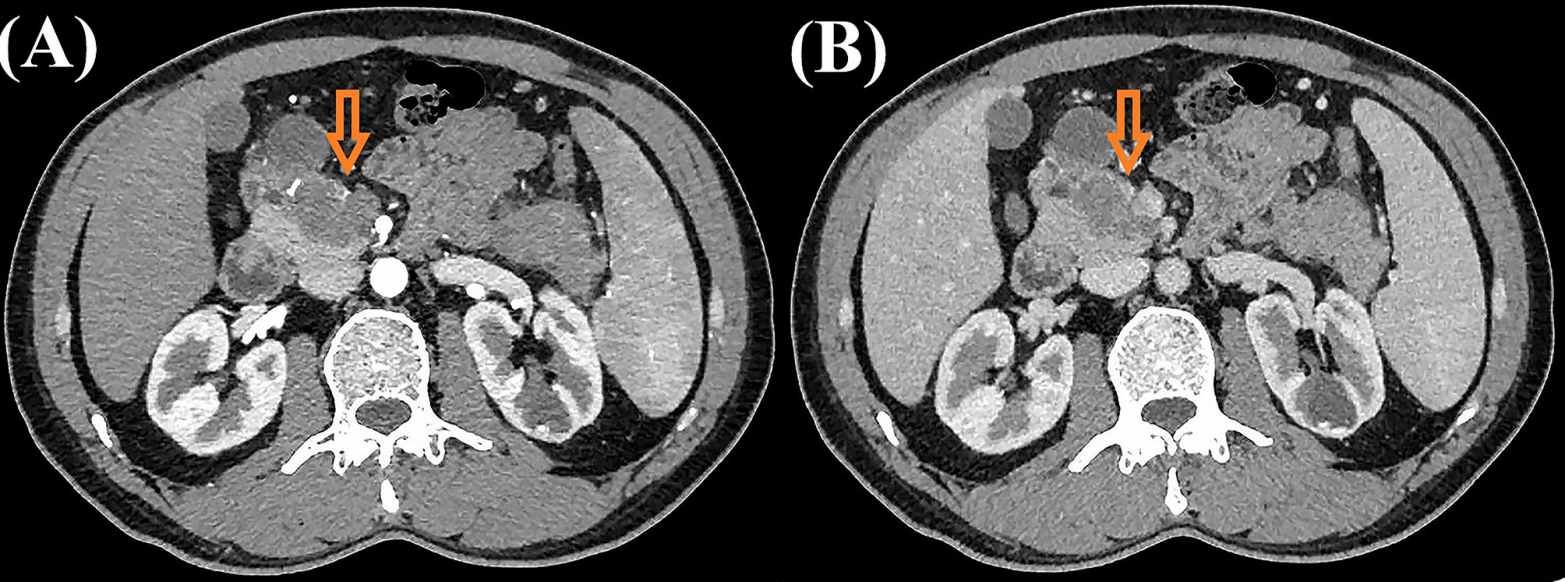
Contrast-enhanced computed tomography images of the patient. Contrast-enhanced computed tomography showed both hypo-enhancement masses in the arterial phase [**(A)**, orange arrow)] and venous phase [**(B)**, orange arrow)].

**Figure 3:**
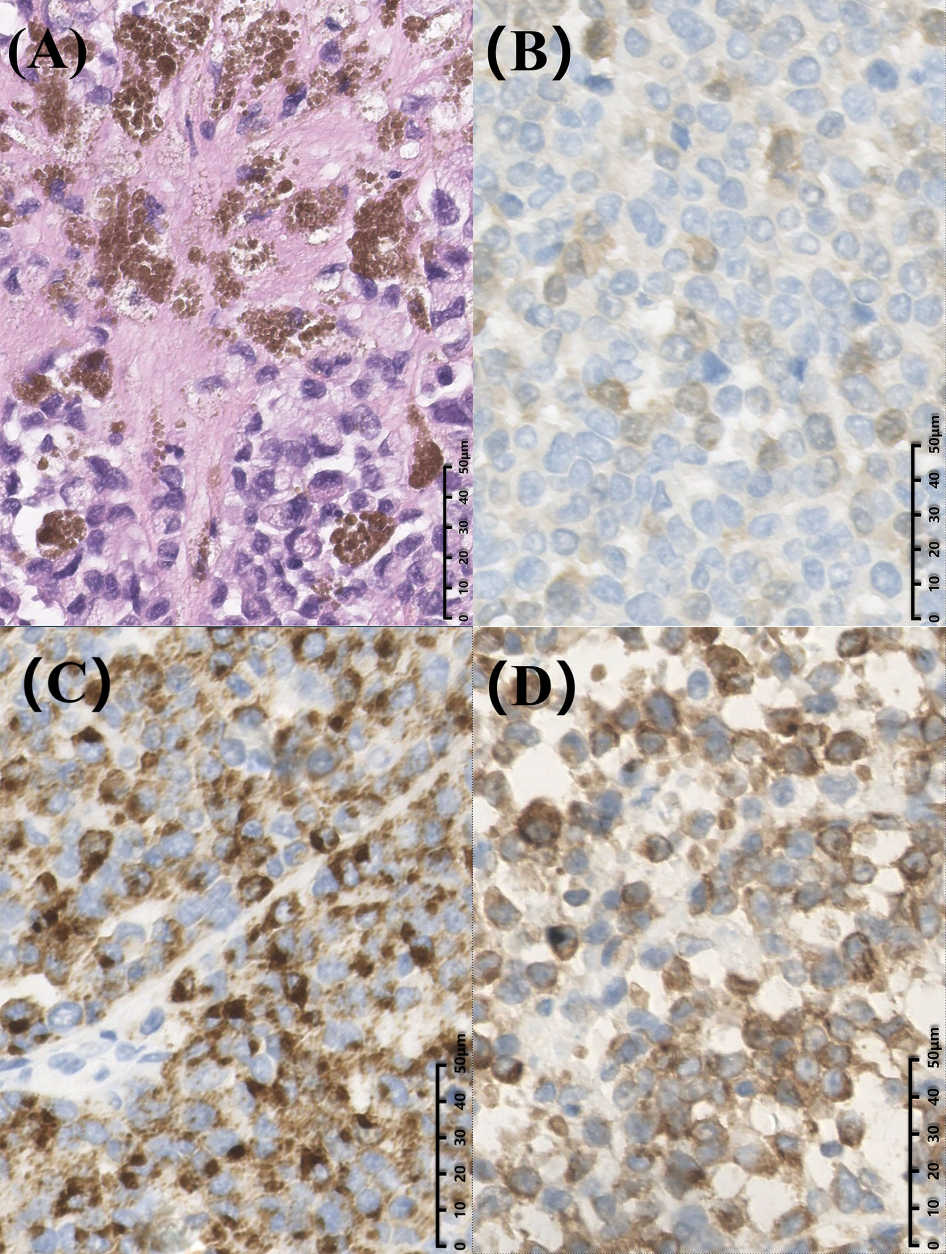
Postoperative histopathological images of the patient. **(A)** Hematoxylin and eosin staining showed some cytoplasm with deep brownish-black granules (magnification, × 400). Immunohistochemical staining displayed S100(+) **(B)**, Human Melanoma Black 45(+) **(C)** and MART-1(+) (magnification, × 400) **(D)**.

A 43-year-old male patient was admitted to our hospital 1 month ago due to unexplained left abdominal distension and pain. Since the onset of the disease, the patient did not have nausea, vomiting, fever or jaundice. He experienced a weight loss of 7 kg over the course of the disease. There was no personal or family history of acute or chronic disease. No abnormal pigmentation of the skin or sclerae or enlarged superficial lymph nodes were observed. The patient showed no tenderness, rebound tenderness or muscle tension on abdominal palpation. The tumor markers carcinoembryonic antigen, carbohydrate antigen 125 and carbohydrate antigen 19-9 were within normal limits. Blood tests for liver and kidney function, electrolyte levels, and coagulation all demonstrated normal results. The patient underwent conventional ultrasound and CEUS examination by an ultrasound system (IU22, Philips Medical Solutions; Mountain View, CA, United States) equipped with a C5-1 abdominal convex transducer (frequency range of 1-5 MHz). Conventional ultrasound demonstrated an abnormal shape, large volume and uneven parenchymal echo of the pancreas. Meanwhile, three hypoechoic lesions were found at the head of the pancreas, the junction of the body and the tail of the pancreas, and the tail of the pancreas, with sizes of approximately 3.3x3.1 cm, 2.4x2.1 cm and 5.4x2.8 cm, respectively (**Figures 1A, B**). Hypoechoic masses partially filled in the splenic vein behind the pancreatic body (**Figure 1B**). All of these lesions had a slightly clear margins, and no obvious signal of blood flow was observed. Then, the patient underwent CEUS with the patient’s consent for further diagnosis. A 2.4-ml ultrasound contrast agent SonoVue (Bracco, Milan, Italy) suspension was injected through the left cubital vein followed by a flush with 5 ml saline. In the CEUS, all of these lesions showed iso-enhancement to slight hypo-enhancement in the arterial phase (**Figure 1C**) and hypo-enhancement in the venous phase (**Figure 1D**). Masses in the splenic vein also showed hypo-enhancement. Chest and abdominal CECT also showed hypo-enhancement in both the arterial and venous phases of these lesions (**Figures 2A, B**), with splenic vein emboli and no other lesions were found. CEUS and CECT imaging features both suggested that the pancreatic lesions were malignant tumors. Based on clinical symptoms, imaging findings and incidence of pancreatic tumors, the patient’s clinical diagnosis was pancreatic carcinoma. Total pancreatectomy and splenectomy and peripancreatic neurectomy with portal vein reconstruction were performed for the patient. Postoperative pathology confirmed a malignant tumor of the pancreas and some cytoplasm with deep brownish-black granules (**Figure 3A**), which invaded the fat, nerves and blood vessels outside the pancreas. Immunohistochemically, the tumor cells were positive for melanocytic marker S-100 (**Figure 3B**), Human Melanoma Black 45 (**Figure 3C**) and MART-1(**Figure 3D**). The above pathological findings revealed malignant melanoma. Four months postoperatively, the patient underwent whole-body CECT examination, which revealed multiple liver, lung metastases. The patient was then given gemcitabine combined with cisplatin chemotherapy and PD-1 immunotherapy with ominous details. The patient and his family gave up treatment because of persistent fever during chemotherapy and fever symptoms even after changing drugs. The patient died 11 months after surgery.

## Discussion

Melanoma is characterized by uncontrolled proliferation of melanocytes, and cutaneous melanoma accounts for 91.2% of all melanomas (2). Non-cutaneous forms of primary melanoma include ocular and mucosal lesions and represent 5.2% and 1.3% of all melanomas, respectively (2). Most melanomas identified in the pancreas are metastatic, and primary pancreatic melanoma is extremely rare and has rarely been discussed. Combined with medical history, physical examination, and imaging, we did not identify the primary site of our patient, so we thought this might be a case of primary pancreatic melanoma.

We reviewed the literature from 2000 to 2021 and found 21 publications regarding the imaging features of pancreatic melanomas (3–23). The clinical findings and imaging features of these 21 reported cases are summarized in **Table 1**. Finally, 26 patients were included for further analysis. The age of the patients ranged from 32 to 75 years, with equal number of men and women. Most of the patients had a clear history of primary melanoma, while the primary lesion search was not performed or not found in some patients. The major symptoms found in these patients were abdominal pain, jaundice, and weight loss, while some patients had no apparent symptoms. Pancreatic melanoma is mainly distributed in the head and tail of the pancreas, with a few diffusely distributed in the pancreatic parenchyma. The tumor marker CA-199 was described in six cases, of which five were normal and one was slightly elevated. Most patients were treated with pancreatectomy, while a few were treated with surgery combined with chemotherapy and immunotherapy. Due to the difference of primary site, time in pancreatic lesion detection and treatment, the survival of patients is different to some extent.

**Table 1:** Pancreatic melanoma in the literature between 2000 and 2021.

Author, year	Age/sex	Primary site	Symptoms	Location	Imaging Findings	Treatment	Follow up(month)	Outcome
DeWitt et al., 2003 (3)	33/M	ND	Epigastric discomfort	Head	US: a 3.3 cm ill-defined mass, dilatation of the extrahepatic bile duct and slight dilatation of the pancreatic duct	Cholecystectomy, palliative gastrojejunostomy and chemical celiac splanchnicectomy	6	Dead
					CT: a 4 cm mass, dilatation of the intrahepatic and extra hepatic biliary and pancreatic ductal dilatation			
					ERCP: a 2 cm malignant-appearing stricture of the distal common bile duct with proximal intrahepatic and extrahepatic biliary dilatation			
	83/F	ND	Abdominal pain	Tail	EUS: a 25 x 20 mm round, cystic mass	Refused medical or surgical treatment	10	Alive
Vagefi et al., 2009 (4)	57/F	Ocular melanoma	Abdominal pain	Tail	CT: hypodense foci	En bloc laparoscopic resection of the distal pancreas and spleen	ND	ND
					MRCP: focal pancreatic enlargement involving the tail with indistinct borders, and associated splenic vein thrombosis			
					PET-CT: FDG-avid uptake			
He et al., 2010 (5)	39/M	Eyeball melanoma	Back pain	Tail	CT: a pseudocyst with a diameter of 7 cm and poor demarcation from surrounding tissue	Melanoma resection with combined distal pancreatectomy and splenectomy, chemotherapy	25	Alive
					ERCP: a deviated main pancreatic duct with no branching			
Lanitis et al., 2010 (6)	69/M	Superficial spreading melanoma	Painless jaundice	Head	CT: a mass with associated intra and extrahepatic biliary tract dilatation	Pancreatoduodenectomy	30	Alive
Mehrotra et al., 2010 (7)	55/M	Unknown primary	Pancreatitis	Head	CT: a 1.5 cm hypodense lesion suspicious of evolving pseudocyst	Pylorus preserving Whipple resection	ND	ND
Portale et al., 2011 (8)	43/F	Cutaneous melanoma	Follow-up	Tail	CT: a hypodense lesion with peripheral enhancement	Distal pancreatectomy with splenectomy	ND	ND
					US: a hypoechoic tumor appropriately 17 mm with a peripheral halo and faint vascular signals in the peripheral part			
					PET: accumulation of the radiotracer in the distal portion of the pancreas and in lymph nodes around vessels of the spleen			
Sperti et al.,2011 (9)	45/M	Unknown primary	Follow-up	Body	CT: a 2 cm hypodense area with dilation of the main pancreatic duct	Distal pancreatectomy with splenectomy, chemotherapy	24	Dead
					MRI: moderate enlargement of the pancreatic head, and a well-circumscribed neoplasm in the body of the pancreas			
Goyal et al., 2012 (10)	47/F	Right arm melanoma	Abdominal pain and jaundice	Distal bile duct	ERCP-assisted biopsy revealed melanoma of the distal bile duct and the ampulla of Vater	Pylorus-preserving pancreaticoduodenectomy, interleukin-2 therapy, oral temozolomide supplemented with thalidomide	15	Dead
	73/F	Left shoulder melanoma	Anorexia and fatigue, jaundice	head	CT: a 4 cm mass	Pylorus-preserving pancreaticoduodenectomy	3	Dead
	58/F	Unknown primary	Abdominal pain and bloating	Head	CT: a 10 x 8 cm mass with additional involvement of the descending duodenum and the distal ileum	Pylorus-preserving pancreaticoduodenectomy, small bowel resection	11.4	Dead
	28/F	Right shoulder melanoma	Abdominal distension	Head	PET-CT: a lesion (2 cm in greatest dimension) centered on the distal common bile duct extending on to the head of the pancreas	Pylorus-preserving pancreaticoduodenectomy with Braun jejunostomy	4.5	Dead
	69/M	Unknown primary	Pancreatitis	Tail	CT: a 4.5 cm mass, a 2.5 cm mass in the hilum of the spleen, and thickening of a portion of the gastric wall	Distal pancreatectomy and splenectomy, total gastrectomy, and Roux-en-Y esophagojejunostomy, chemotherapy	26	Dead
Sugtmoto, et al., 2013 (11)	46/M	Nasal Cavity	Follow-up	Body	PET-CT: significant fluorodeoxyglucose accumulation with a maximum standardized uptake value of 10.6	Distal pancreatectomy with splenectomy as well as regional lymphadenectomy, chemotherapy	10	Dead
					CT: a 33 x 31 mm oval tumor with a clear boundary and prolonged enhancement			
Larsen et al., 2013 (12)	32/F	Cutaneous malignant melanoma	Abdominal pain, itching, and jaundice	Head	CT and a transabdominal ultrasonography-assisted biopsy: a large malignant melanoma metastasis (5 cm in greatest dimension)	Interleukin-2 and interferon-α, radiotherapy, pancreaticoduodenectomy	18	Alive
Birnbaum et al., 2013 (13)	45/F	Dorsal melanoma	Epigastric pain, weight loss	Head	EUS: a 50 mm tumor without vascular invasion	pancreatectomy	72	Alive
Jana et al., 2015 (14)	75/M	Right chest malignant melanoma	Routine surveillance	Head, body	MRI: a 1.7 cm focal pancreatic mass and a large left upper retroperitoneal lymph node	Stereotactic gamma knife radiosurgery, immunotherapy	ND	ND
					PET-CT: increased metabolic activity in the proximal pancreas, with standardized uptake value of 10.36			
					EUS: several hypoechoic, rounded, well-defined masses. The dominant lesion was a 24.6 x 21.4 mm mass in the body of the pancreas			
De et al., 2016 (15)	58/F	Uveal malignant melanoma	Jaundice and abdominal pain	Head	CT: a low-attenuating lesion measuring 4 x 3 cm and a thinner section of the distal bile duct suspicious for compression	Duodenopancreatectomy, chemotherapy	ND	ND
					EUS: a solid, heteroechoic with predominantly hypoechoic areas, well-defined lesion with regular contours (measuring 3.1 x 2.6 cm)			
Ben et al., 2017 (16)	55/F	Unknown primary	Upper abdominal pain and itching	Head	CT and MRI: a 6 x 5 cm mass with peripheral enhancement, leading to intra and extrahepatic biliary tract dilatation	Pancreaticoduodenectomy	15	Dead
Liu et al., 2018 (17)	54/M	Cutaneous melanoma	Follow up	The junction of pancreatic head and uncinate	CT: a solid hypovascular mass measuring approximately 3.1 x 2.4 cm compressed the lower common bile duct resulting in expansion of the upstream bile ducts	Laparoscopic pancreaticoduodenectomy	6	Alive
Nakamura et al., 2019 (18)	67/F	Left nasal cavity	Abdominal pain	Tail	CT: a rounded, well-defined lesion with low attenuation and pancreatic ductal dilatation surrounded by a high-attenuation rim	Distal pancreatectomy	24	Alive
					MRI: the center of the mass was hyperintense on T1 and hypointense on T2, and the diffusion weighted image showed a hyperintense peripheral rim of the mass			
					ERCP: smooth narrowing and displacement of the pancreatic duct with upstream dilatation			
					EUS: a 35 mm mass, hypoechoic and heterogeneous with central anechoic areas			
					CE-EUS: iso-enhancement during the 20 s phase and hypo-enhancement during the 120 s phase of the peripheral rim of the mass with central non enhancement			
					PET-CT: intense FDG uptake			
Voudoukis et al., 2020 (20)	62/M	A scalp lesion	Painless jaundice	Pancreatic parenchyma	CT: diffuse heterogeneity in the head of the pancreas, and slight enlargement of the pancreatic body and tail, but no discrete pancreatic lesion described	Dabrafenib (a BRAF inhibitor) and trametinib (a MEK inhibitor)	4	Alive
					EUS: pancreatic parenchyma diffusely enlarged, many hypoechoic well-demarcated lesions all with low blood flow signal			
Jin et al., 2020 (19)	43M	Unknown primary	Epigastric pain	Diffusion distribution	Contrast-enhanced ultrasound: multifocal hypoechoic mass in the pancreas	Extended total pancreatectomy together with portal vein reconstruction and extensive lymphadenectomy, interferon-alpha 2b therapy	20	Alive
					CT: multifocal lesions with low density and the largest one had a diameter of 2.6 cm			
					Three-dimensional vascular reconstruction of CT: a tumor thrombus formed in the superior mesenteric vein and central segment of the splenic vein			
					MRI: multi focal masses, hypointense on T1 and isointense on T2, heterogeneously enhanced during the arterial phase and portal venous phase			
Vargas et al., 2021 (21)	60/M	ND	Abdominal pain, jaundice, and pruritus, weight loss	Head	US: significant dilation of the intra and extrahepatic bile ducts and a mass in the pancreas	ND	ND	ND
					CT: a 3.6 x 4.2 cm mass, round, well-defined, with no calcifications or documentation of ganglia or vessel involvement, no pancreatic gland atrophy or main pancreatic duct dilation			
					MRI: a 3.4 x 3.5x 4.4 cm mass with hyperintense enhancement on T1			
					EUS: a hypoechoic, heterogeneous mass			
Zeman et al., 2021 (23)	72/M	Unknown primary	Jaundice	Head	CT: head of the pancreas enlarged and had a diameter anteroposteriorly of 33 mm with a prominence towards the duodenum	ND	ND	Dead
Shamim et al., 2021 (22)	38/F	Right eye choroidal melanoma	Intermittent abdominal pain and vomiting	Head	CT: an ill-defined heterogenous lesion measuring approximately 2.2 x 2.3 cm	Classic Whipple procedure and segmental transverse colectomy	ND	ND
					PET-CT: ill-defined heterogenous lesion in the junction of head and neck of the pancreas, increased tracer uptake			

ND, Not described; US, Grayscale ultrasound; CT, Computed tomography; MRI, Magnetic resonance imaging; MRCP, Magnetic resonance cholangio pancreatography; EUS, Endoscopic ultrasonography; ERCP, Endoscopic retrograde cholangiopancreatography; PET-CT, Positron Emission Computed Tomography.

Unfortunately, no specific imaging features for pancreatic melanomas have been found at present. Although some imaging modalities, such as ultrasonography, computed tomography, nuclear magnetic resonance imaging, endoscopic ultrasound, and positron emission tomography, are valuable to some extent, it remains a challenge to diagnose pancreatic melanomas preoperatively if there is no clear history of primary melanoma. Conventional ultrasound was performed in 4 of the 26 patients, and it usually presented as a hypoechoic mass with or without dilation of the bile duct and pancreatic duct. CT was performed in 21 of the 26 patients, and most of the findings were hypoechoic solid masses with or without dilatation of the bile duct and pancreatic duct, and only two of them presented pseudocysts. CECT findings were described in three cases, two with peripheral enhancement and one with delayed enhancement. Endoscopic ultrasound was performed in six cases, most of which presented as well-defined hypoechoic solid masses, and only one presented as cystic mass. There have been a few reports of other imaging techniques for diagnosing pancreatic melanomas, such as magnetic resonance imaging, magnetic resonance cholangiopancreatography, endoscopic retrograde cholangiopancreatography and positron emission computed tomography. None of these imaging methods revealed specific features for pancreatic melanomas.

In this patient, the symptoms of abdominal distension and pain and weight loss were similar to those in the literature. CEUS and CECT indicated the pancreatic lesions were malignant tumors. In addition, pancreatic ductal adenocarcinoma (PDAC) is the most common form of pancreatic carcinoma, which accounts for the majority (90%) of pancreatic neoplasms (24–26). Therefore, based on the clinical symptoms, imaging findings and incidence of pancreas neoplasms, the surgeons misdiagnosed it as pancreatic carcinoma. ​It is crucial for us to accurately distinguish PDAC from pancreatic melanomas, which determines the patients’ treatment options and the doctors’ management of patients. Carbohydrate antigen 19-9 levels are elevated in 80% of pancreatic cancer patients (27). However, in this patient, the tumor markers carcinoembryonic antigen, carbohydrate antigen 125, and carbohydrate antigen 19-9 were within normal limits. We can also see some differences between pancreatic melanomas and PDAC in ultrasound images. PDACs are derived from epithelial cells that line the pancreatic duct (28), and most PDACs are localized in the pancreatic head (29). Consequently, the typical imaging features of PDAC in conventional ultrasound are hypoechoic mass, dilatation of the pancreatic duct, and dilatation of the bile duct (30). And PDAC is generally a solitary poorly defined lesion (31). Nonetheless, in this case, the large size of the pancreatic head lesion did not cause dilation of the main pancreatic duct. And the margins of these lesions were more clearly relative to PDAC. In the CEUS, PDAC is typically significantly hypo-enhancing in the arterial phase, because of the desmoplastic reaction with low vascular density that is present in 90% of cases (32–38). However, the lesions of this patients showed iso-enhancement to slightly hypo- enhancement in the arterial phase. Another common solid pancreatic neoplasm pancreatic neuroendocrine tumors typically present as hyper-enhancing lesions in the arterial phase of CEUS examinations (39–41). So when we encounter a solid lesion of the pancreas like this patient with a different sonographic appearance than typical PDAC and pancreatic neuroendocrine tumor, the tumor markers are also not significantly elevated. We should consider other rare tumor possibilities, there is no doubt that pancreatic melanoma is one of the possibilities. It is important for clinicians to consider a broad differential diagnosis when faced with inconclusive imaging studies of pancreatic tumors. Endoscopic ultrasound-guided fine-needle aspiration (EUS-FNA) plays an important role in providing cytological confirmation for diagnosis (14). In the literature we reviewed, 9 of 26 patients underwent EUS-FNA and all were confirmed melanoma, which demonstrated the important role of EUS-FNA in the differential diagnosis of pancreatic tumors. In addition, EUS-FNA is now the gold standard method for sampling the pancreas (15). If conditions permit, patients can be clearly diagnosed by EUS-FNA.

In summary, it is difficult to correctly diagnose pancreatic melanoma before surgery, especially if there is no history of primary lesions. The sonographic features of pancreatic melanoma in our patient are different from common solid lesions PDAC and pancreatic neuroendocrine tumors, but more cases are needed to summarize and validate these findings. Preoperative EUS-FNA could be considered for further confirmation, and preoperative biopsy combined with comprehensive imaging examination will contribute to identifying adequate surgical candidate (42, 43). Hence, awareness of pancreatic melanoma from imaging features and tumor markers may aid in the management of patients.

## Data availability statement

The raw data supporting the conclusions of this article will be made available by the authors, without undue reservation.

## Ethics statement

Written informed consent was obtained from the individual(s) for the publication of any potentially identifiable images or data included in this article.

## Author contributions

ZY performed the literature review and wrote the manuscript. HY and WL supported the data collection and manuscript revision. YL supervised the writing and revision of the manuscript. All authors contributed to the article and approved the submitted version.

## Funding

This research was supported by National Natural Science Foundation of China, No. 82071940.

## Conflict of interest

The authors declare that the research was conducted in the absence of any commercial or financial relationships that could be construed as a potential conflict of interest.

## Publisher’s note

All claims expressed in this article are solely those of the authors and do not necessarily represent those of their affiliated organizations, or those of the publisher, the editors and the reviewers. Any product that may be evaluated in this article, or claim that may be made by its manufacturer, is not guaranteed or endorsed by the publisher.
